# P02.108. Effects of integrative medicine on pain and anxiety for inpatient orthopedic patients

**DOI:** 10.1186/1472-6882-12-S1-P164

**Published:** 2012-06-12

**Authors:** N Ghildayal, P Johnson, A Fyfe-Johnson, J Dusek

**Affiliations:** 1 Allina Hospitals & Clinics, Center for Healthcare Innovation, Minneapolis, USA; 2 Penny George Institute for Health and Healing, Abbott Northwestern Hospital, Minneapolis, USA

## Purpose

To examine the effects of integrative medicine (IM) on pain and anxiety in an inpatient orthopedic population.

## Methods

Data were obtained from the electronic health record (EHR) for selected orthopedic inpatient admissions during 2010. Four orthopedic populations were identified by APR-DRG categories: 1) hip replacement, 2) knee replacement, 3) back surgery, and 4) medical back pain. Receipt of IM therapies was the treatment of interest. Primary outcomes were change in patient reported pain and anxiety. Patients rated pain and anxiety levels before and after IM treatments using a self-report intensity scale (0 to 10), with higher scores indicating higher levels of pain and anxiety. Chi-square tests were used to test for significant differences in the percentage of patients who received specific IM therapies. Significant differences in mean pain and anxiety score changes were evaluated using paired t-tests.

## Results

Overall, there were 2,866 patients with a hip replacement, knee replacement, or back pain/surgery DRG. Of these, 1,013 (35.3%) received at least one IM visit. Of those, 584 (57.7%) had a single therapeutic treatment. Overall, 63.1% of the patients received acupuncture alone or in combination with another therapy, and 24.7% received Korean hand therapy alone or in combination. The most common single treatment was acupuncture (39.7%). All DRG groups experienced significant reductions in pain and anxiety after IM treatment. Knee replacement patients reported the greatest pain reduction, with a decrease of 1.7 points (58.4% reduction), on average, after receiving IM treatment (p<0.001). Back surgery patients reported the greatest post treatment difference in anxiety scores among DRG groups, with a mean decrease of 1.4 points (70.8% reduction) (p<0.001).

## Conclusion

Orthopedic patients reported lower pain and anxiety scores after receiving integrated therapies. Further research should be done to evaluate the efficacy of specific IM interventions on pain and anxiety within an orthopedic patient population.

